# Maximal mouth opening capacity: percentiles for healthy children 4–17 years of age

**DOI:** 10.1186/1546-0096-11-17

**Published:** 2013-04-22

**Authors:** Lukas Müller, Hubertus van Waes, Christoph Langerweger, Luciano Molinari, Rotraud K Saurenmann

**Affiliations:** 1 Department for Orthodontics and Pediatric Dentistry, University of Zurich, Zurich, Switzerland; 2 Public School Dental Services in the City of Zurich, Zurich, Switzerland; 3 Department of Growth and Development, Children’s Hospital, University of Zurich, Zurich, Switzerland; 4 Department for Pediatric Rheumatology, Children’s Hospital, University of Zurich, Zurich, Switzerland

**Keywords:** Diagnosis, Arthritis, Juvenile idiopathic Arthritis, Outcome research

## Abstract

**Background:**

A reduced mouth opening capacity may be one of the first clinical signs of pathological changes in the masticatory system. The aim of this retrospective cross-sectional study was to create age related percentiles for unassisted maximal mouth opening capacity (MOC) of healthy children.

**Methods:**

All recordings of MOC as measured at the yearly dental examinations of school children in the city of Zurich, Switzerland, between August 2009 and August 2010 were extracted from the database. The program LMSchartMaker Pro Version 2.43, Huiqi Pan and Tim Cole, Medical Research Council, 1997–2010 was used to calculate age and sex related reference centiles.

**Results:**

Records from 22^′^060 dental examinations were found during the study period. In 1286 (5.8%) the maximal interincisal measurement was missing. Another 55 examinations were excluded because of missing data for sex (7), age at examination (11) or because the value was deemed to be pathologically low (37). Thus, a total of 20^′^719 measurements (10^′^060 girls, 10^′^659 boys) were included in the analysis. The median age (range) was 9.9 years (3.3-18.3) for girls and 10.0 years (2.8-18.7) for boys. The mean MOC (range) was 45 mm (25–69) for girls and 45 mm (25–70) for boys. Age related percentiles were created for girls and boys separately, showing the 3^rd^, 10^th^, 25^th^, 50^th^, 75^th^, 90^th^, and 97^th^ percentile from 3 through 18 years of age.

**Conclusions:**

In these 20^′^719 unselected school children MOC increased with age but showed a wide range within children of the same age.

## Background

Measurement of maximal mouth opening capacity (MOC) reflects mandibular range of motion. It is a simple but important clinical parameter for follow-up and outcome assessment of diverse affections of the stomatognathic system, e.g. odontogenic infections [1], temporomandibular disorders (TMD) [2], trauma [3,4] and tumors [5].

In children with juvenile idiopathic arthritis (JIA) the temporomandibular joint (TMJ) is affected in about 50% [6-8], but diagnosis and treatment are often delayed because of lack of symptoms in early TMJ arthritis [7]. A reduced MOC may be one of the first clinical signs of TMJ involvement [6,9,10].

A limited mouth opening is part of widely accepted assessment tools for the function of the masticatory system such as the Helkimo’s Clinical Dysfunction Index [11], the Craniomandibular Index [12] and Research Diagnostic Criteria for Temporomandibular Disorders [13,14]. These tools are built for the use in adults and use a cut-off value for the definition of reduced mouth opening capacity. For children only few data about normal values of MOC exist. A single cut-off value does not seem adequate for the definition of limited MOC in growing individuals. Furthermore, most existing datasets are of limited value because they are based on too small numbers of participants or do not cover the entire age range. Despite these limitations, the existing normal values [15-25] are pointing towards an influence of growth on the maximal MOC and a wide range of normalcy within a certain age category.

The aim of our study was to create age related percentiles for the maximal mouth opening capacity of healthy children and adolescents which may serve as a basis for clinical evaluation and research projects.

### Subjects and methods

#### Subjects

As part of the local health service system, all schoolchildren (95% of European descent) in the city of Zurich, Switzerland, have to undergo mandatory yearly dental examinations which are free of charge for the families. Parents can choose to have their children examined by the school dental service or at their private dentist. During the past three years an average of 81 percent of children in the city of Zurich have attended the school dental service for these examinations.

Mouth opening capacity (MOC), i.e. the unassisted maximal interincisal distance was included as part of the routine dental examinations of school children starting from August 17^th^ 2009 (start of the school year).

## Methods

**Figure 1:**
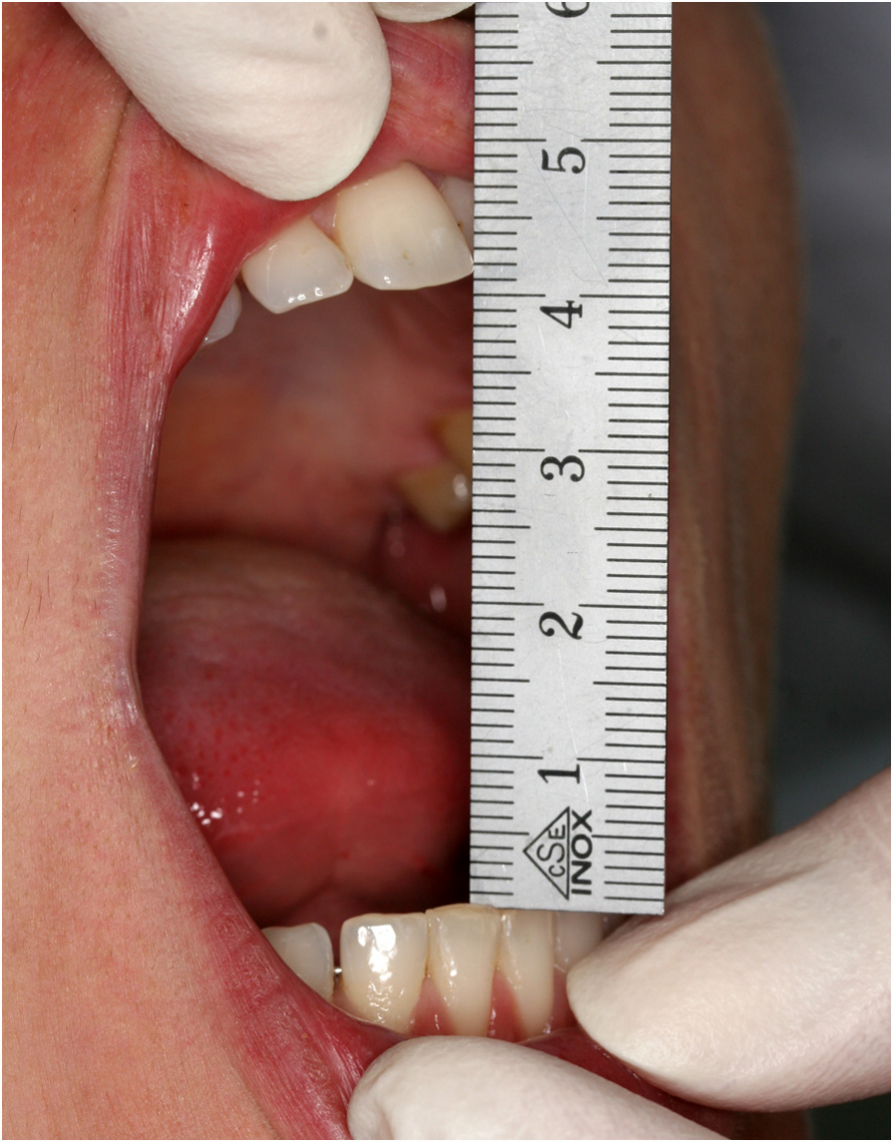
**Measuring the maximal mouth opening capacity.** Measuring the maximal mouth opening capacity with a metallic calliper (millimetre scale) between the incisal edges of upper and lower central incisors.

Maximal mouth opening capacity (MOC) was defined as the maximal interincisal distance on unassisted active mouth opening. The measurements were taken at the end of the annual dental examination, after the children had to open their mouths widely several times. This served as mobilizing exercise. For the measurement the children were verbally encouraged to open their mouths as far as possible. A metallic ruler with a millimetre scale was passively placed between the edges of upper and lower central incisors (Figure 1). The measurement was read and recorded to the nearest millimetre. If the central incisors were missing or the patient was not cooperative, no measurement was performed. In case of erupting central incisors the pair with the smaller interincisal distance was chosen. In order to make the measurement simple and quick for daily practice, positive overbite values were not taken into account. Negative overbite values (an open space between the incisors in closed mouth position) were measured perpendicularly to the occlusal plane and subtracted from the measured interincisal distance. The measurements were entered into the school dental services database.

### Reliability measurements

As part of the quality assessment at the public school dental services 41 children were measured repeatedly: the first time at the end of the routine examination as usual (CL1); a second measurement was obtained approximately 30 minutes after the first measurement by the same dentist (CL2). The same group was then measured a third time by a different dentist (LM) approximately 10 minutes later on the same day in a different room. The involved dentists did not have any contact with each other or with each other’s results and both were blinded for the previous measurements.

On July 9th 2010 (end of the school year) the following data were extracted from the database: maximal interincisal distance, age at examination and sex. The data were extracted in an anonymous way in conformity with the rules of our institutional review board.

### Statistics

The data were analysed using the program LMSchartMaker Pro Version 2.43, Huiqi Pan and Tim Cole, Medical Research Council, 1997–2010 [26].

Descriptive statistics for the clinical measurements and for the differences between the repeated measurements were performed using the JMP 9 software from the SAS Institute Inc, Cary NC, USA. To validate the performed measurements, the differences between the repeated measurements were plotted against their average by Intra-class correlation (ICC) as recommended by Bland and Altman [27]. Mean differences, standard errors, intra-and inter-observer correlations were calculated.

## Results

A total of 32 dentists were involved in the acquisition of the data. Records from 22′060 dental examinations were found during the study period.

**Figure 2:**
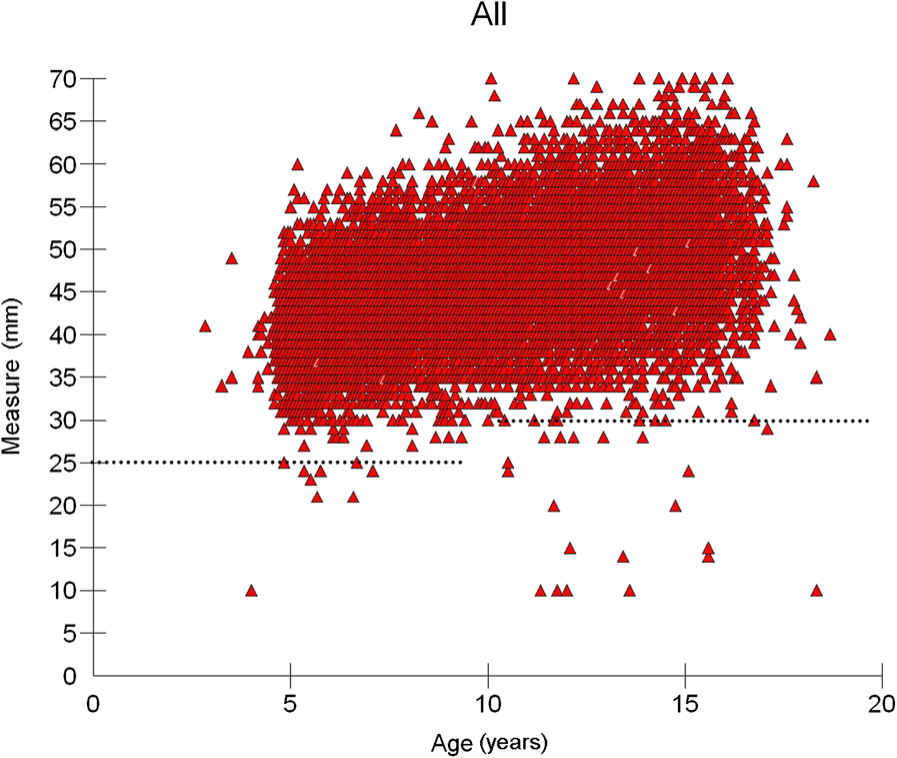
**Scatterplot of all 22′060 measurements with outlayers.** Extreme outliers (below dotted line) were defined as values less than 25 mm in 3–9 year-old children and less than 30 mm in children older than 10 years.

In 1286 (5.8%) records the measurement of MOC was missing because of missing central incisors or lack of cooperation. Another 18 examinations were excluded because of missing data for gender (7) or age at examination (11). Based on the scatterplot of all measurements (Figure 2) extreme outliers were defined as values less than 25 mm in 3–9 year-old children and less than 30 mm in children older than 10 years. Thus another 37 examinations were eliminated from the evaluation. Finally, a total of 20′719 measurements (10′060 girls, 10′659 boys) were included in the analysis. The number of individuals in every age group for boys and girls are displayed in Table 1.

**Table 1:** Number of individuals in every age group for boys and girls with MOC values (mm): mean, 10th and 90th percentile

**Age**	**2y**	**3y**	**4y**	**5y**	**6y**	**7y**	**8y**	**9y**	**10y**	**11y**	**12y**	**13y**	**14y**	**15y**	**16y**	**17y**	**18y**	**Total**
n: male	1	2	166	1249	1146	1159	687	889	967	895	894	848	852	685	198	19	2	10659
mean MOC (mm)	-	-	40.1	40.7	42.2	43.6	44.4	45.5	46.1	47.3	48.5	49.2	51.2	51.6	52.0	51.2	-	45
10^th^ Percentile			35	35	36	37	38	39	40	40	41	42	43	43	43	42	-	25-70
90^th^ Percentile			46	46	48	50	51	52	53	55	56	57	60	60	61	60		
n: female	0	2	200	1285	1024	1130	610	810	915	901	811	784	800	648	127	12	1	10060
mean MOC (mm)	-	-	40.4	40.7	41.8	43.2	44.0	45.3	46.2	47.6	48.1	48.9	48.8	49.4	49.0	47.4	-	45
10^th^ Percentile			35	35	36	37	38	39	40	40	41	42	42	42	41	36	-	25-69
90^th^ Percentile			45	46	48	49	51	52	53	55	55	56	56	56	58	58		

The median age (range) was 9.9 years (3.3-18.3) for girls and 10.0 years (2.8-18.7) for boys. The mean MOC (range) was 45 mm (25–69) for girls and 45 mm (25–70) for boys. Up to the age of 13 years the mean MOC values for the individual age groups were not significantly different for girls and boys. For the age groups of 14 to 17 years increasingly higher mean MOC values were measured for boys in comparison to girls: 2.2 mm to 3.8 mm (Table 1).

**Figure 3:**
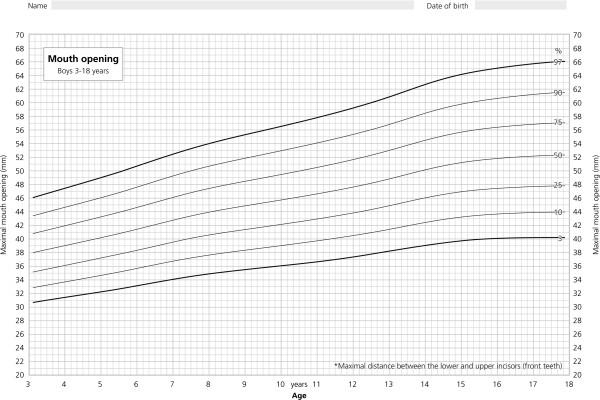
**Age related percentiles for boys.** Percentiles of maximal mouth opening capacity for boys showing the 3rd, 10th, 25th, 50th, 75th, 90th, and 97th percentile from 3 through 18 years of age.

**Figure 4:**
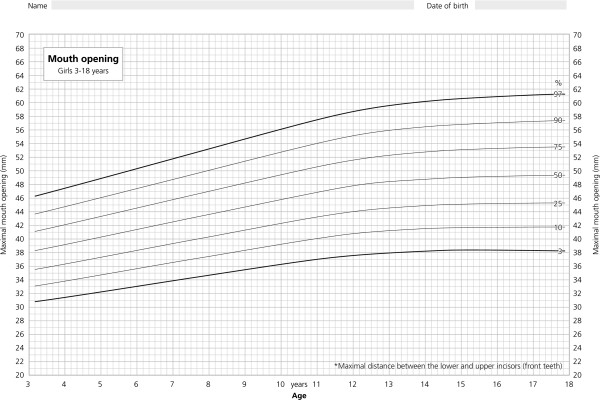
**Age related percentiles for girls.** Percentiles of maximal mouth opening capacity for girls showing the 3rd, 10th, 25th, 50th, 75th, 90th, and 97th percentile from 3 through 18 years of age.

The dataset was entered into the LMS program for the creation of age related percentiles. Best results were achieved using the following settings: L = 1, i.e. L constant, set to L = 0.65 by the program, M = 5, S = 2 for girls, and L = 1, set to 0.5 by the program, M = 7, S = 2 for boys. Age related percentiles for MOC in boys and girls are given in Figure 3 and 4.

### Reliability measurements

The group of 41 children (mean age 8.7, range 5.2-17.4) with repeated measurements contained 23 girls. The mean values measured increased from CL1 (mean 45.9 mm) to CL2 (mean 46.7 mm) and further increased to LM (mean 47.3 mm). The mean absolute difference between CL1 and CL2 was 1.6 mm (median 2 mm, range 0-5 mm, SD 1.3 mm), between CL1 and LM 1.6 mm (median 1 mm, range 0-6 mm, SD 1.2 mm) and between CL2 and LM 1.4 mm (median 1 mm, range 0-6 mm, SD 1.4 mm).

### Intra-observer reliability

Paired comparison of the 2 measurements of observer 1 resulted in a mean difference of 0.8 mm (p-value: 0.013) with a standard error (SE) of 0.30 and a correlation of 0.97.

### Inter-observer reliability

Paired comparison of the first measurement of observer 1 with the measurement of observer 2 resulted in a mean difference of 1.4 mm (p-value < 0.0001; SE 0.24) and a correlation of 0.98, whereas the comparison of the second measurement of observer 1 with the measurement of observer 2 resulted in a mean difference of 0.6 mm (p-value: 0.0504; SE 0.30) and a correlation of 0.97.

## Discussion

To our knowledge this data sample of 20′719 unselected school children is the largest sample reported so far and covers the entire age range where unassisted mouth opening can be measured [15-23]. We believe that these percentiles will therefore be of great importance for future studies as well as clinical assessment of children with affections of the masticatory system.

MOC increased smoothly with age but showed a wide range within children of the same age group. As far as results can be compared, this is in line with other studies [15-18,22,23]. Landtwing [15] recorded MOC in a group of 976 individuals (age range 5–19 years) and observed a slight increase of the median value from 43 mm to 55 mm for 5 and 18 year olds, respectively. In every age group he found a considerable range of MOC starting with 34 mm to 55 mm for 5 year old children and ending with 36 mm to 65 mm for 18 year old adults. The same characteristic of a small but significant increase of MOC with age accompanied by large ranges in every age group was observed by Hirsch et al. [16] who assessed a sample of 1011 children (age range of 10–17 years). The slight increase of MOC with age in children and adolescents is partly explained by mandibular growth. Growth results in increasing mandibular length which geometrically influences the linear interincisal measurements [17,28-30]. This is supported by the observation of Landtwing [15]: in his sample MOC had a better correlation with stature height (0.69) than with chronological age (0.66) of the children.

Having the effect of mandibular length in mind, the wide range of MOC within every age group can be explained not only by inter-individual TMJ mobility but also by differences in craniofacial morphology and skeletal age [31]. That’s why Dijkstra et al. [32] recommend using linear measurements of MOC longitudinally within the same subject over time. This way, the bias caused by inter-individual differences of mandibular morphology can be overcome.

Because of the wide inter-individual range, we cannot recommend to use a certain cut-off value for the assessment of MOC in children with potential affection of the masticatory system. Also, we do not believe that these percentiles will be able to solve the dilemma of interpretation of a single MOC measurement. However, we hope that they will become an important tool for the longitudinal follow-up of children with a high risk for TMJ affections, e.g. children with juvenile idiopathic arthritis. To our knowledge, our results make it possible for the first time to take into account the influence of chronological age when judging MOC from early childhood to late adolescence.

For the age group up to 13 years the percentiles for MOC didn’t differ between boys and girls. In accordance with girls reaching puberty earlier, a slight flattening of the percentiles can be observed after the age of 12 years (Figure 4). The annual increase in MOC of males declines later after the age of 14 to 15 years and ends up with slightly higher MOC values for all percentiles (Figure 3). This can be explained by the male “growth spurt” which starts later, results in higher growth rates and lasts longer. In addition the setting of parameter M = 7 (M = 5 for females) in the LMS program also has influenced the percentile curves for males to look more bumpy. Our observation is in line with the observation of List et al. [23] who examined a sample of 862 children with an age-range from 12 to 18 years. Several other studies reported higher values for MOC in males [15,16,22,33,34]. Hirsch et al. [16] observed a significant difference of 1.9 mm with larger opening in male subjects (age group 10-17 years). Although statistically significant, it has to be questioned whether these differences are clinically relevant.

When we performed the MOC examination repeatedly, the mean values increased significantly from first to second and second to third measurements. This is in line with a study in adults by Hesse et al. [35] who found it necessary to open the mouth maximally more than four times in females and three times in males before levelling off to a consistent maximum value was observed. The differences between the measurements of CL1, CL2 and LM are therefore rather a display of a mobilizing effect on the TMJ than presenting solely intra- or inter-observer variability. The measurements in our study were carried out only once. A “warm-up” procedure to mobilize the TMJ by performing MOC three times and taking the highest value would have increased the measured MOC and the reliability of the results [35,36]. But as the MOC was the last parameter examined, the children got at least some “warm-up”-exercises by opening widely during the dental examination.

Our study is limited by the fact that the Zurich population is 95% Caucasian. Ethnicity was not recorded in our sample and we can therefore not entirely exclude the possibility that ethnicities were represented slightly differently in our sample than in the overall Zurich population. Nevertheless our data reflect findings of a predominant Caucasian population. Also, only 81% of school children attend the school dental services. Although we do not have any reasons to believe that the remaining 19% were entirely different in body dimensions, we cannot completely exclude a possible bias. Another limitation is the fact that the measurements were carried out in different places of the public school dental services of Zurich. A total of 32 dentists were involved in the acquisition of the data. The inter-observer reliability was tested only for two different observers (both co-authors of this study). This fact may have minimized a possible difference between the measurements. The inter-observer reliability was acceptable and comparable with the reliability observed by List et al. [23].

One may also consider the fact that we did not correct for overbite a limitation of this study. However, admitting to this concern one should consequently not only consider overbite but also overjet as factors influencing MOC. It is evident that both a large overbite and a large overjet will geometrically increase the interincisal distance measured during unassisted MOC. Taking these considerations into account, we believe that such a complicated assessment would no longer fulfil the requirement of an easy and quick tool for everyday use.

Due to the retrospective character of the study no systematic questionnaire or clinical TMJ-examination were performed to exclude individuals with TMD. Controversial results are found in the literature to whether the influence of TMD on MOC is negligible or not [23,24,37,38]. Diagnoses with limitation of jaw motion according to the Research Diagnostic Criteria for TMD (i.e. muscle pain with limited opening, disc displacement without reduction with limited opening) are rare in this age group [22,23,33,34]. Therefore it can be assumed that these conditions do not significantly influence population “normal values” of jaw motion.

## Conclusion

MOC increases with age but shows a wide range within children of the same age.

That’s why we recommend individual follow up of MOC according to the published percentiles in children with a high risk for TMJ affections, e.g. children with juvenile idiopathic arthritis. To achieve the optimal maximal MOC values, a warm-up procedure has to be included into the measurement process.

## Consent

Written informed consent was obtained from the patient's parent for publication of any accompanying images.

## Abbreviations

MOC: Maximal mouth opening capacity; TMD: Temporomandibular disorders; JIA: Juvenile idiopathic arthritis; TMJ: Temporomandibular joint; LMS: L: λ (the power in the Box-Cox transformation for “correcting” the skewness); M: μ (median); S: σ (a coefficient of variation); PC: Percentile; SE: Standard error.

## Competing interests

The authors declare that they have no competing interests.

## Authors’ contributions

LM conceived of the study, and participated in its design and was involved in data collection and wrote the manuscript. HvW participated in the design of the study and was involved in data collection and reviewing the manuscript. CL participated in the design and coordination of the study and was involved in data collection and helped to review the manuscript. LMo participated in the design of the study and performed the statistical analysis and reviewed the manuscript. RKS conceived of the study and its design, participated in the statistical analysis and writing of the manuscript. All authors read and approved the final manuscript
